# Does vaccination with 4CMenB convey protection against meningococcal serogroup B strains not predicted to be covered by MATS? A study of the UK clonal complex cc269

**DOI:** 10.1080/21645515.2019.1688039

**Published:** 2019-12-06

**Authors:** Maria Stella, Maria Giuliani, Alessia Biolchi, Sara Tomei, Rosita De Paola, Xilian Bai, Ray Borrow, Jay Lucidarme, Rita La Gaetana, Daniela Toneatto, Mariagrazia Pizza, Laura Serino, Elena Mori, Marzia Monica Giuliani

**Affiliations:** a GSK, Siena, Italy; b Meningococcal Reference Unit, Public Health England, Manchester, UK

**Keywords:** *Neisseria meningitidis*, meningococcus serogroup B, meningococcal B vaccine, 4CMenB, meningococcal antigen typing system, serum bactericidal antibody assay

## Abstract

The Meningococcal Antigen Typing System (MATS) has been developed as an hSBA surrogate to evaluate potential coverage afforded by the 4-component meningococcal serogroup B vaccine (4CMenB: *Bexsero*, GSK). We investigated whether the lower value of MATS coverage among invasive Meningococcus serogroup B clonal complex 269 strains from the United Kingdom (53% in 2014–2015 versus 73% in 2007–2008) reflected the lower bactericidal activity of the vaccine against these isolates. A total of 34 MATS-negative strains (31 were cc269 or closely related) were tested against pooled sera from 32 or 72 4CMenB-vaccinated infants in a serum bactericidal antibody assay in presence of human complement (hSBA). All infants had received four 4CMenB doses in the first 2 y of life. Baseline sera comprised 180 pooled samples from healthy-unvaccinated 2-month-old infants. Twenty of the 34 (59%) MATS-negative strains were killed in hSBA with titers ≥4 by pooled sera from vaccinated infants. There were 13/34 strains with hSBA titers ≥4 and at least a 4-fold rise in titer with respect to pooled baseline sera, and 10/34 with hSBA titers ≥8 and at least a 4-fold rise in titer with respect to baseline. These data confirm MATS as a conservative estimate for predicting strain coverage by 4CMenB.

Vaccination is the primary strategy of protection against invasive meningococcal disease (IMD), which is characterized by a relatively high mortality rate even when treated appropriately. Among meningococcal serogroups, serogroup B (MenB) predominates in Europe and in many industrialized countries worldwide that employ national serogroup C vaccination programs.^1,2^ 4CMenB (*Bexsero*, GSK) is a multi-component MenB vaccine containing three recombinant proteins (variant 1.1 of factor H binding protein [fHbp], peptide 8 variant 2/3 of Neisseria adhesin A [NadA], and peptide 2 of Neisserial Heparin Binding Antigen [NHBA]) together with an outer membrane vesicle (OMV) derived from an outbreak strain from New Zealand (OMV NZ) and expressing Porin A (PorA) P1.4 as the major antigen.^3^

4CMenB is licensed in more than 40 countries for infants from 2 months of age. The United Kingdom (UK) was the first country to introduce 4CMenB into the National Immunization Program in September 2015, from which the first data on the population impact of 4CMenB vaccination were obtained. The first estimate of the effectiveness of two doses of 4CMenB in preventing MenB IMD in infants was 82.9% (95% confidence interval [CI] 24.1–95.2).^4^

The serum bactericidal antibody assay in the presence of human complement (hSBA) is used to measure the immunogenicity of meningococcal vaccines. An hSBA titer ≥4 is recognized as a surrogate marker of protection.^5^ hSBA cannot be used on a large scale to evaluate strain panels, especially in infants from whom blood sample volumes are minimal. The Meningococcal Antigen Typing System (MATS) is a vaccine antigen-specific sandwich ELISA which has been developed as an hSBA surrogate and has been shown to be a conservative method to evaluate potential coverage of 4CMenB. MATS measures the expression levels and immunologic cross-reactivity of vaccine antigens in a given MenB strain.^6,7^

We recently evaluated temporal trends in MATS coverage of IMD strains from the UK in 2014–15 compared with 2007–08.^8^ The three most represented clonal complexes (cc) in the UK in both time periods were cc269, cc41/44, and cc213.^8^ We observed a decrease in MATS coverage for cc269 in 2014–2015 (53%) compared to 2007–08 (73%), while cc41/44 retained very high MATS coverage (94% in both time periods), and cc213 showed an increase in coverage (17% in 2007–2008 and 23% in 2014–2015).^8^ In this work, we investigated whether the observed lower MATS values result in an underestimation of coverage of strains isolated in the UK in 2014–15, by designing a study in which pooled sera from infants vaccinated with 4CMenB were tested by hSBA against a subpanel of MATS-negative strains, mainly belonging to, or closely related to, cc269. Isolates from 2007 to 2008 were not retested.

The characteristics of 251 isolates from all culture-confirmed cases of MenB IMD from the UK between 2014 and 2015 have been previously described.^8^ Of these 251 strains, 86 were negative in MATS (defined as having relative potency less than the positive bacterial threshold for fHbp, NHBA or NadA; or lacking PorA P1.4) for all four vaccine antigens. Strains belonging to cc269 mostly express NHBA peptide 17 in conjunction with fHbp subvariant 2.19, or 1.13; or NHBA peptide 21 in conjunction with fHbp subvariant 1.15. A subpanel of 34 strains, negative in MATS for all vaccine antigens, harboring NHBA peptide 21 or 17, and harboring fHbp subvariant 2.19, 1.13 or 1.15 was selected for additional testing in hSBA. Most of these 34 strains belonged to cc269 (26/34), the others belonged to cc35 (3/34) and unassigned cc (5/34). The five unassigned isolates belonged to sequence types (ST-4713, ST-8061, ST-11306, ST-11307 and ST-11469) that have >5 MLST loci in common with the subgroup founder ST, ST-275 (cc269), but are officially excluded from cc269 by virtue of having only three (i.e. less than the minimum of four) loci in common with the founder ST, ST-269 (Figure 1a, b, Supplement Table S1). Baseline pooled sera comprised 180 samples from healthy-unvaccinated 2-month-old infants who were enrolled in study V72P13 (NCT00657709) conducted in Finland, the Czech Republic, Germany, Austria, and Italy.^9^ 4CMenB coverage of the 34 MATS-negative strains was evaluated in hSBA using pooled sera from randomly selected 4CMenB-vaccinated infants. Two pools of post-immunization sera were obtained from 32 to 72 infants who received a 4-dose series of 4CMenB in study V72P12_E1 (NCT00721396, NCT00944034) conducted in Belgium, UK, the Czech Republic, Germany, Italy, and Spain.^10,11^ All infants received 4CMenB in a 2, 4, 6 months schedule with booster during the second year of life (12, 18 or 24 months of age). Pooled sera comprised samples collected approximately 1 month after the last 4CMenB dose.

**Figure 1.**
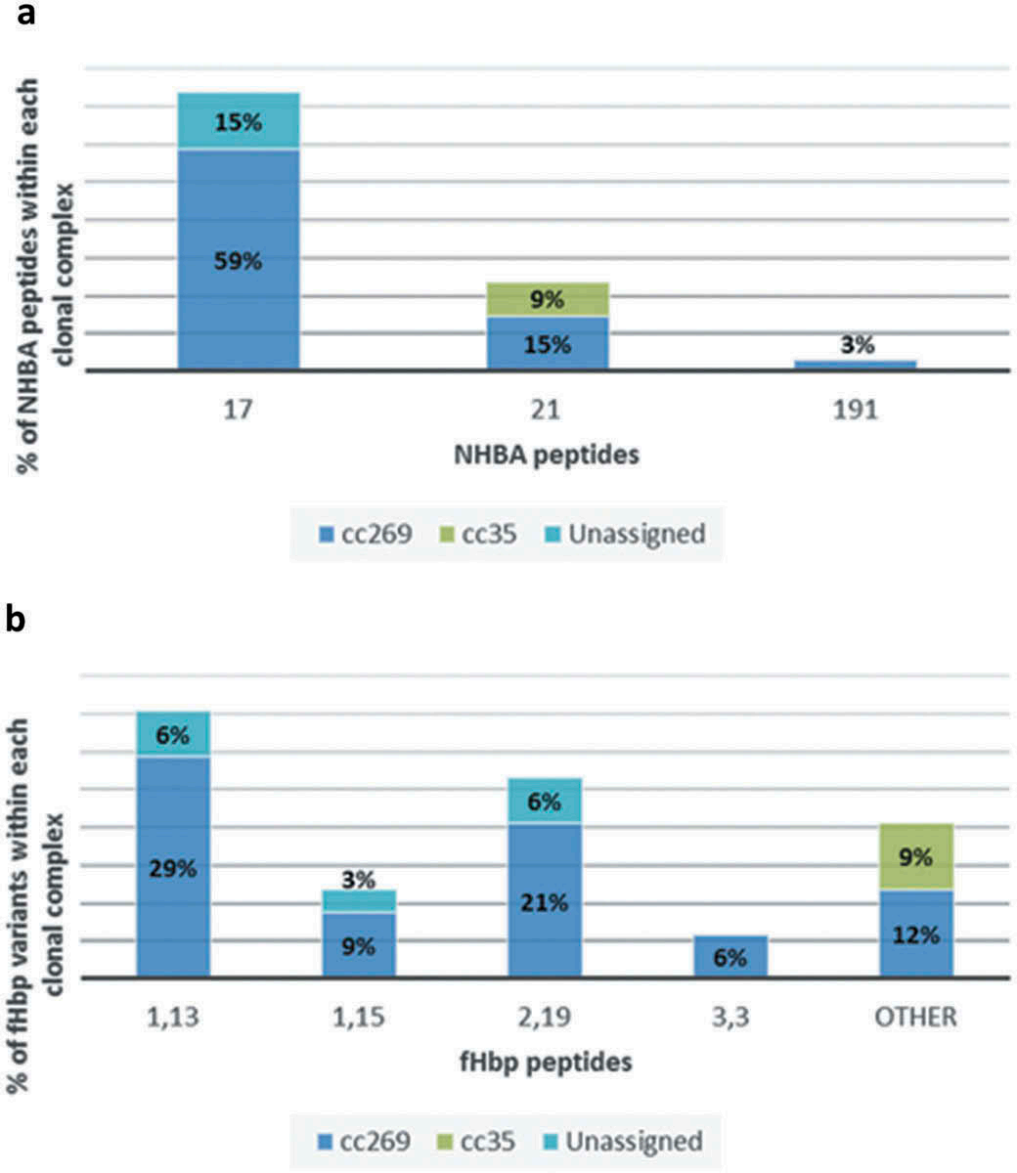
Distribution of NHBA peptides and fHbp variants within clonal complexes of the 34 MATS-negative strains tested in hSBA. NHBA, Neisserial Heparin Binding Antigen; fHbp, factor H binding protein. Tabulated data are provided in the Supplement Table S1.

hSBA assays were performed as described by Borrow et al. with minor modifications.^12^ MenB bacteria were sub-cultured overnight on Chocolate Agar, re-suspended in Mueller Hinton Medium to an optical density (OD) of 0.05 and grown until OD of 0.25 before use in the assay. hSBA titers were determined as the last dilution that resulted in at least a 50% reduction in colony forming units (CFU) relative to the number of CFU present in the reaction without serum. Human plasma obtained from volunteer donors under informed consent was selected for use as complement source with a particular MenB strain only if it did not significantly reduce CFU of that strain relative to T0 when added to the assay at a final concentration of 50%. The final assay mixture contained 25% human plasma.

Twenty out of 34 MATS-negative strains were killed in hSBA with titers ≥4 by pooled sera collected from infants immunized with four doses of 4CMenB (Figure 2). There were a further 13/34 strains with hSBA titers ≥4 and at least a 4-fold rise in titer with respect to pooled baseline sera, and 10/34 with hSBA titers ≥8 and at least a 4-fold rise in titer with respect to baseline.

**Figure 2.**
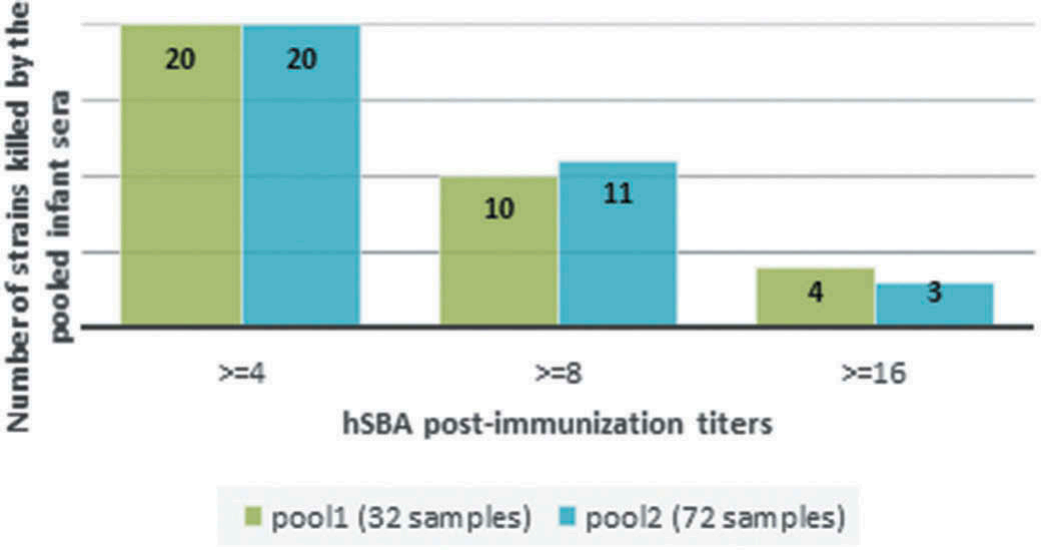
34 strains assessed for killing in hSBA using pooled sera from infants one-month post-last 4CMenB vaccination (34 MenB strains negative in MATS for all four vaccine antigens. hSBA categories are mutually exclusive).

Experiments with large panels of sera have established a correlation between bactericidal titers achieved using pooled sera and seroresponse rates in infants.^13^ In the panel of 34 MATS-negative invasive isolates tested, around two-thirds were killed by pooled infant sera in hSBA. The results of our study provide further evidence supporting the hypothesis that MATS is a conservative estimate for predicting strain coverage by 4CMenB.^14^ In MATS, the positive bactericidal threshold used to indicate susceptibility was established by the relationship between MATS and hSBA in pooled sera obtained from infants who received 4 doses of 4CMenB, using a conservative hSBA cutoff of 8.^6^ While positive hSBA assay results are considered predictive of protection against IMD, hSBA levels below the threshold are not necessarily an indication of susceptibility to disease.^15^ Both conditions likely contribute to the tendency for MATS to underestimate coverage. Additionally, MATS is unable to account for the known synergies between antibodies induced by the individual vaccine components.^16,17^

The MenB strains that cause invasive disease differ regionally and evolve continually over time. Regular monitoring of coverage after the implementation of vaccines into routine vaccination schedules is needed to evaluate ongoing vaccine effectiveness. Reductions in strain coverage over time could imply antigenic shift, potentially due to vaccine pressure, that could require changes to the vaccine or the vaccination schedule to maintain the effectiveness of the vaccination program overall. Continued monitoring of MenB clinical isolates contributes to our understanding of the changing epidemiology of MenB disease and the impact of vaccines in different populations.

In conclusion, MATS is a useful tool to analyze large panels of strains and to predict the coverage of 4CMenB against MenB strains causing IMD but is likely to be conservative in its estimation. Apparent changes in MATS vaccine coverage over time warrant ongoing investigation to monitor the effectiveness of 4CMenB vaccination. Further investigation of antibody synergy could help to understand the relationship between the results observed using hSBA versus those obtained in MATS.

## Trademark statement

*Bexsero* is a trademark of the GSK group of companies.

## Data sharing statement

Information on GSK’s data sharing commitments and requesting access to anonymized individual participant data and associated documents can be found at www.clinicalstudydatarequest.com.
