# Supplementary material for: Does vaccination with 4CMenB convey protection against meningococcal serogroup B strains not predicted to be covered by MATS? A study of the UK clonal complex cc269
Source: Hum Vaccin Immunother. 2019 Dec 6;16(4):945–8. doi: 10.1080/21645515.2019.1688039 (PMC7227617; doi:10.1080/21645515.2019.1688039)
Supplement: Supplemental Material [file khvi-16-04-1688039-s001.docx]

**Does vaccination with 4CMenB convey protection against meningococcal serogroup B strains not predicted to be covered by MATS? A study of the UK clonal complex cc269**

**Supplement**

**Table S1** Molecular typing, MATS relative potency of 4CMenB antigens and hSBA titers at baseline and one month post-last vaccination against 34 meningococcal serogroup B strains negative in MATS for all four vaccine antigens.

|  |  |  |  |  | **hSBA titers using pooled infant sera** | | |
| --- | --- | --- | --- | --- | --- | --- | --- |
|  |  | **MATS Relative Potency** | | | V72P13 | V72P12E1_2 | V72P12E1_3 |
| **Isolate** | **Clonal complex** | **NHBA PBT 0.294** | **fHpb PBT 0.012** | **NadA PBT 0.009** | **Pre**  N= 180 | **Post4**  N=32 | **Post4**  N=72 |
| M14-0240382 | Unassigned | 0.137 | 0.002 | 0 | 2 | <2 | 2 |
| M14-0240390 | cc269 | 0.088 | 0.01 | 0 | <2 | 8 | 8 |
| M14-0240451 | cc269 | 0.148 | 0.002 | 0 | <2 | 4 | 4 |
| M14-0240465 | Unassigned | 0.128 | 0.012 | 0 | <2 | 8 | 8 |
| M14-0240466 | Unassigned | 0.106 | 0.011 | 0 | 2 | 4 | 4 |
| M14-0240503 | cc269 | 0.187 | 0.002 | 0 | 2 | 2 | <2 |
| M14-0240507 | cc269 | 0.164 | 0.001 | 0 | 2 | 2 | 2 |
| M14-0240596 | Unassigned | 0.169 | 0.00051 | 0 | <2 | <2 | <2 |
| M14-0240619 | cc269 | 0.15 | 0.002 | 0 | 2 | 4 | 4 |
| M14-0240630 | cc269 | 0.141 | 0.002 | 0 | <2 | <2 | <2 |
| M14-0240640 | cc269 | 0.105 | 0 | 0 | <2 | 4 | 4 |
| M14-0240646 | cc269 | 0.219 | 0.012 | 0 | 2 | 8 | 16 |
| M14-0240647 | cc269 | 0.141 | 0.012 | 0 | 2 | 16 | 8 |
| M15-0240003 | cc269 | 0.202 | 0.001 | 0.003 | 2 | 2 | 2 |
| M15-0240050 | cc269 | 0.077 | 0.001 | 0 | <2 | <2 | <2 |
| M15-0240056 | cc269 | 0.147 | 0.001 | 0 | <2 | 2 | 2 |
| M15-0240076 | cc269 | 0.184 | 0.003 | 0 | 2 | 4 | 4 |
| M15-0240084 | cc269 | 0.119 | 0.012 | 0 | <2 | 2 | <2 |
| M15-0240090 | cc269 | 0.075 | 0.01 | 0 | <2 | 2 | <2 |
| M15-0240122 | cc269 | 0.271 | 0.011 | 0 | 2 | 16 | 16 |
| M15-0240169 | cc269 | 0.229 | 0 | 0 | 2 | 2 | <2 |
| M15-0240196 | cc269 | 0.27 | 0.012 | 0 | <2 | 8 | 8 |
| M15-0240211 | cc269 | 0.073 | 0 | 0 | <2 | 2 | <2 |
| M15-0240226 | cc35 | 0.192 | 0.002 | 0 | 2 | 4 | 4 |
| M15-0240236 | cc35 | 0.158 | 0.001 | 0 | <2 | <2 | <2 |
| M15-0240286 | cc269 | 0.148 | 0.012 | 0 | 2 | 8 | 8 |
| M15-0240325 | cc269 | 0.068 | 0.009 | 0 | 2 | 4 | 4 |
| M15-0240373 | cc269 | 0.181 | 0.012 | 0 | 2 | 16 | 16 |
| M15-0240424 | cc269 | 0.259 | 0.012 | 0 | <2 | 2 | <2 |
| M15-0240437 | cc269 | 0.202 | 0.011 | 0 | <2 | 4 | 8 |
| M15-0240446 | cc35 | 0.169 | 0 | 0 | 2 | 4 | 4 |
| M15-0240466 | cc269 | 0.038 | 0.009 | 0 | 2 | 8 | 8 |
| M15-0240468 | Unassigned | 0.143 | 0.007 | 0 | 2 | 4 | 4 |
| M15-0240476 | cc269 | 0.166 | 0.012 | 0 | 2 | 16 | 8 |

PBT = positive bacterial threshold
